# Sensory domain of the cell cycle kinase CckA regulates the differential DNA binding of the master regulator CtrA in *Caulobacter crescentus*

**DOI:** 10.1016/j.bbagrm.2018.08.006

**Published:** 2018-10

**Authors:** Sharath Narayanan, Lokesh Kumar, Sunish Kumar Radhakrishnan

**Affiliations:** School of Biology, Indian Institute of Science Education and Research, Thiruvananthapuram 695551, Kerala, India

## Abstract

Sophisticated signaling mechanisms allow bacterial cells to cope with environmental and intracellular challenges. Activation of specific pathways ameliorates these challenges and thereby warrants integrity. Here, we demonstrate the pliability of the CckA-CtrA two-component signaling system in the freshwater bacterium *Caulobacter crescentus*. Our forward genetic screen to analyze suppressor mutations that can negate the chromosome segregation block induced by the topoisomerase IV inhibitor, NstA, yielded various point mutations in the cell cycle histidine kinase, CckA. Notably, we identified a point mutation in the PAS-B domain of CckA, which resulted in increased levels of phosphorylated CtrA (CtrA~P), the master cell cycle regulator. Surprisingly, this increase in CtrA~P levels did not translate into a genome-wide increase in the DNA occupancy of CtrA, but specifically enriched its affinity for the chromosomal origin of replication, C_*ori*_, and for a very small sub-set of CtrA regulated promoters. We show that through this enhanced binding of CtrA to the C_*ori*_, cells are able to overcome the toxic defects rendered by stable NstA through a possible slow down in the chromosome replication cycle. Taken together, our work opens up an unexplored and intriguing aspect of the CckA-CtrA signal transduction pathway. The distinctive DNA binding nature of CtrA and its regulation by CckA might also be crucial for pathogenesis because of the highly conserved nature of the CckA-CtrA pathway in alphaproteobacteria.

## Highlights

• The PAS-B domain of the CckA histidine kinase modulates the distinctive DNA binding of CtrA in *Caulobacter crescentus*.
• The DNA binding of CtrA at the C_*ori*_ and at S-phase promoters is specifically enhanced due to a mutation in the PAS-B domain.
• Plasticity in the CckA-CtrA pathway facilitates *Caulobacter* to overcome the NstA-induced chromosome segregation defect.

## 1 Introduction

Bacteria harbor robust signaling mechanisms to tolerate stressful conditions. Exquisitely fine-tuned regulatory cascades in bacteria impart their effect to regulate development in response to changes in the internal or external milieu. The aquatic α‑proteobacterium, *Caulobacter crescentus* (henceforth *Caulobacter*), has emerged as a powerful model organism for studying the complex signaling mechanisms that control cell cycle and development in response to environmental cues. During its cell cycle, *Caulobacter* undergoes asymmetric division to produce progenies with distinct developmental fates. One of the daughter cells, the swarmer cell, is motile and its locomotion is assisted by the polar flagellum [1,2]. In contrast, the stalked daughter cell is sessile and capable of replicating its chromosome [3,4]. The G1-like swarmer cell has to terminally differentiate into a stalked cell to enter into the proliferative phase. This G1 to S-like transition is marked by the shedding of the flagellum, retraction of the pili, and production of a stalk at the same cell pole.

In the swarmer cells, the master transcriptional regulator, CtrA, inhibits the DNA replication. The *Caulobacter* origin of replication, C_*ori*_, is bound by CtrA, which prevents replisome formation in the swarmer cells [5]. Concurrent with the swarmer to stalked cell transition, CtrA is degraded by the ClpXP protease [6] thus allowing the binding of DnaA, the replication initiator, to the C_*ori*_ triggering chromosome replication [7]. Apart from blocking DNA replication initiation, CtrA also serves as a transcription factor to drive the expression of numerous developmentally important genes in a cell cycle-dependent manner [8].

**Fig. 1:**
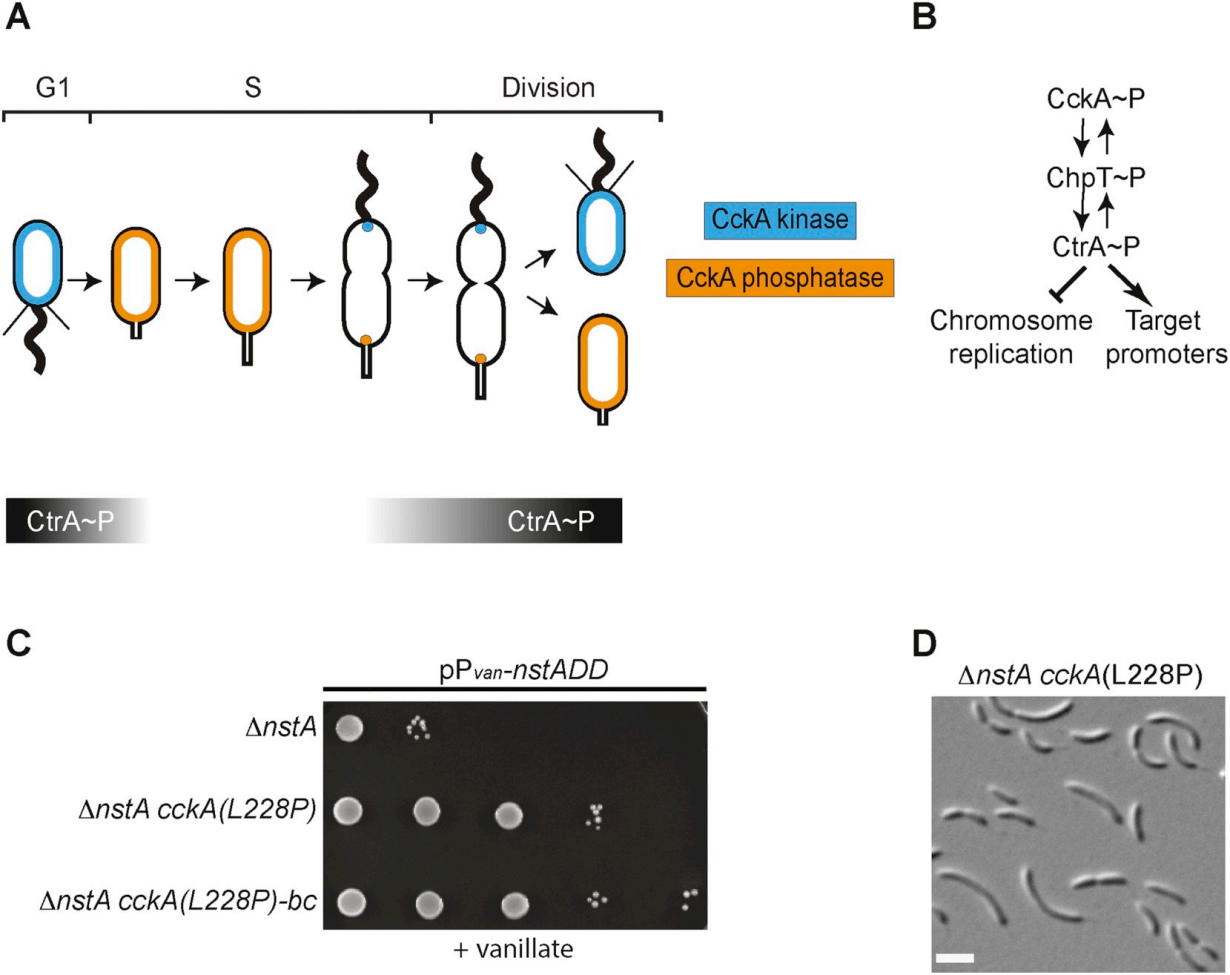
Cell cycle regulation in *Caulobacter crescentus* by the CckA-CtrA pathway. (A) Schematic representation of the dual switching of CckA between the kinase mode (blue) and the phosphatase mode (orange) in the swarmer and the stalked cell compartments, respectively. The graded bars indicate the time during which CtrA (black) is present during the cell cycle. (B) The bidirectional flow of phosphate between CckA, ChpT and CtrA. In the swarmer cells, CckA transfers the phosphate group to the phosphotranferase, ChpT, which further donates the phosphate to CtrA. The active phosphorylated form of CtrA (CtrA~P) can bind to various target promoters of several cell cycle regulated genes, as well as repress the initiation of chromosome replication. (C) Growth of ∆*nstA* cells overproducing NstADD, and harboring either *wild*-*type cckA* or *cckA*(L228P) or *cckA*(L228P) mutation back-crossed into a clean ∆*nstA* background [∆*nstA cckA*(L228P)-*bc*]. Cells, as indicated, were diluted five folds and spotted on media containing 0.5 mM vanillate. (D) Differential interference contrast (DIC) image of the suppressor mutant, ∆*nstA cckA*(L228P). Scale bar: 2 μm.

The differential activity of CtrA in the swarmer and stalked cells is of paramount significance for generating different cell fates. Multiple levels of regulation involving control at the level of synthesis, stability, and activity exist for the regulation of CtrA during cell cycle [9,10]. The phosphorylated form of CtrA (CtrA~P) represents the active form that binds to DNA [11]. The phosphorylation of CtrA is catalyzed by an essential hybrid cell cycle histidine kinase/phosphatase, CckA, which phosphorylates CtrA through the single domain histidine phosphotransferase, ChpT (Fig. 1A and B) [[12], [13], [14], [15]]. CckA gets autophosphorylated and it eventually transfers the phosphate group *via* ChpT to the master regulator, CtrA. In the swarmer, and pre-divisional cells, the kinase activity of CckA ensures the abundance of active CtrA~P, while in the stalked cell compartment, the phosphatase activity of CckA is predominant ensuring the dephosphorylation, and degradation, of CtrA (Fig. 1A and B) [16]. The N-terminus of the CckA protein has two transmembrane helices and also contains two distinct sensory Per-ARNT-Sim domains, PAS-A and PAS-B [17,18]. The catalytic core of CckA comprises of a DHp (dimerization histidine phosphotransfer) domain, which is the site of histidine autophosphorylation, and an ATP binding catalytic assisting domain [19,20]. The C-terminal receiver domain in CckA shuttles the phosphate group to CtrA, through the ChpT phoshotransferase [21]. The PAS-A domain regulates density-dependent CckA kinase activity and its subcellular accumulation at the cell poles. The second PAS domain, PAS-B is needed for targeting CckA to the new cell pole and for cyclic-di-guanylate (*c*-di-GMP) stimulated CckA phosphatase activity [[22], [23], [24], [25]].

Recent evidence have shown that in addition to developmental regulatory proteins such as CtrA, the cell cycle progression in *Caulobacter* is controlled by a cytoplasmic redox fluctuation [26]. We had shown that a redox-dependent regulator, NstA, whose activation is coupled to the cytoplasmic redox state, inhibits the DNA decatenation activity of topoisomerase IV (Topo IV) during the early stages of cell cycle [26]. Apart from the cytoplasmic redox control of NstA activity, additional layers of regulation for NstA exist at the level of transcription by the transcription factors, GcrA and CcrM, and at the level of protein abundance by the ClpXP protease. A stable version of NstA, NstADD, is resistant to protein degradation by ClpXP. Overproduction of NstADD from an inducible promoter induces lethality in *Caulobacter* [26]. In this study, we investigated the regulatory networks that possibly fine-tune NstA activity *in vivo*. Towards this, we exploited the lethality induced by NstADD, to conduct an unbiased forward genetic screen to analyze extragenic suppressor mutation(s) that can negate NstADD toxicity. Strikingly, through this screen, we have identified suppressor mutations in *cckA* that influences the DNA binding activity of CtrA in a distinctive manner. We show that the CckA(L228P) mutation enhances the CtrA~P levels. Surprisingly, the increase in CtrA~P levels does not result in an increase of CtrA binding to all CtrA binding regions on the chromosome. The DNA binding of CtrA is specifically increased only at the C_*ori*_ and a very small sub-set of CtrA dependent promoters. Finally, we show that the enhanced binding of CtrA to the C_*ori*_ rescues the toxicity caused by NstADD by possibly slowing down the chromosome replication process to compensate for the slowed down segregation caused by the inhibitory effects of NstADD on the Topo IV.

## 2 Materials and methods

### 2.1 Growth conditions and media

*Caulobacter* strains were grown on rich PYE media (0.2% peptone, 0.1% yeast extract, 1 mM MgSO_4_, 0.5 mM CaCl_2_) or M5G media (M5G low-phosphate medium: 10 mM PIPES, pH 7, 1 mM NaCl, 1 mM KCl, 0.05% NH_4_Cl, 0.01 mM Fe/EDTA, 0.2% glucose, 0.5 mM MgSO_4_, 0.5 mM CaCl_2_ and 0.05 mM phosphate) [27,28], and incubated at 29 °C, unless specifically mentioned. The *Caulobacter* strains were subjected to electroporation, øCr30 mediated transductions, and intergeneric conjugations (using *E*. *coli S17-1*) as previously described [[29], [30], [31]]. *E*. *coli* strains, EC100D (Epicentre, WI, USA), and S17-1 were grown on LB media and incubated at 37 °C, unless specifically mentioned.

### 2.2 *In vivo* phosphorylation

*In vivo* phosphorylation experiments were performed as described previously [32]. Briefly, single colony of cells picked from a PYE agar plate was washed with M5G medium lacking phosphate and was grown overnight in M5G with 0.05 mM phosphate to an optical density of 0.3 at 660 nm. One milliliter of culture was labeled for 4 min at 28 °C using 30 μCi of γ-[^32^P]ATP. Upon lysis, proteins were immunoprecipitated with a polyclonal CtrA (34) or a polyclonal CckA (15) antisera using Protein A agarose (Roche, Switzerland) and the precipitates were resolved by SDS–polyacrylamide gel electrophoresis. The radiolabelled CtrA or CckA were quantified using ImageJ [33] and the values were normalized to the relative total protein levels of CtrA or CckA, in each strain, as determined by immunoblot of the lysates.

### 2.3 Extragenic suppressor screen

∆*nstA* or *WT* cells were UV irradiated with 700 and 900 × 100 μJ/cm^2^ energy using CL 1000 UV Cross linker (UVP, Cambridge, UK). The irradiated cells were diluted into PYE media followed by 6 h incubation. The cells were then electroporated with the plasmid pBVMCS-4-P_*van*_-*nstA*DD and plated on PYE supplemented with gentamycin and vanillate inducer. Individual colonies were then grown in liquid PYE containing gentamycin, and vanillate inducer for overnight. Two criteria were used to confirm the extragenic mutation: (i) plasmids from the selected mutants were again transformed into *WT Caulobacter* and checked for *nstA*DD toxicity, to avoid the possibility that the suppression is due to any mutations on the plasmid, and (ii) the plasmid cured mutants were retransformed with fresh pBVMCS-4-P_*van*_-*nstA*DD, to confirm that the toxicity suppression is indeed due to a mutation in the chromosome. Mutations were mapped by next generation sequencing on an Illumina platform at Fasteris, Switzerland.

### 2.4 Backcross

The *cckA* backcross plasmid, pSN155 (pNPTS138-*cckA*-backcross conferring kanamycin resistance) was integrated by homologous recombination nearby the *cckA* gene in the Δ*nstA cckA*(L228P) mutant, SN208. Following this, ΦCr-30-mediated generalized transduction was used to transfer the mutant *cckA* allele from SN208 into a clean *WT* or Δ*nstA* strain. The strains that had lost the pSN155 plasmid, after allele exchange, were selected using sucrose counter-selection. The presence of *cckA*(L228P) mutation in the backcrossed strains was confirmed by sequencing of the *cckA* gene.

### 2.5 ChIP-Seq

Chromatin Immunoprecipitation (ChIP) experiments were carried out as described earlier [31]. Mid-log phase cells were cross-linked in 10 mM sodium phosphate (pH 7.6) and 1% formaldehyde at room temperature for 10 min and on ice for 30 min thereafter, washed thrice in phosphate buffered saline (pH 7.4) and lysed in 5000 Units of Ready-Lyse lysozyme solution (Epicentre Technologies, WI, USA). Lysates were sonicated on ice using 7 bursts of 30 s to shear DNA fragments to an average length of 0.3–0.5 kbp. The cell debris were cleared by centrifugation at 14,000 rpm for 2 min at 4 °C. Lysates were normalized by protein content, wherein 500 μg equivalent protein was taken and diluted up to 1 mL using ChIP buffer (0.01% SDS, 1.1% Triton X-100, 1.2 mM EDTA, 16.7 mM Tris-HCl [pH 8.1], 167 mM NaCl plus protease inhibitors [Complete™ EDTA-free, Roche, Switzerland]), and pre-cleared with 80 μL of protein-A agarose (Roche, Switzerland) saturated with 100 μg BSA. Ten % of the supernatant was removed and used as total chromatin input DNA for qPCR analyses. To the remaining supernatant, anti-CtrA [34] antibody was added (1:500 dilution), and incubated overnight at 4 °C. Immuno complexes were trapped with 80 μL of protein-A agarose beads pre-saturated with BSA. The beads were then washed once each with low salt buffer (0.1% SDS, 1% Triton X-100, 2 mM EDTA, 20 mM Tris-HCl [pH 8.1], 150 mM NaCl), high salt buffer (0.1% SDS, 1% Triton X-100, 2 mM EDTA, 20 mM Tris-HCl [pH 8.1], 500 mM NaCl) and LiCl buffer (25 mM LiCl, 1% NP-40, 1% sodium deoxycholate, 1 mM EDTA, 10 mM Tris-HCl [pH 8.1]), and twice with TE buffer (10 mM Tris-HCl [pH 8.1], 1 mM EDTA). The protein·DNA complexes were eluted in 500 μL freshly prepared elution reagent (1% SDS, 1 mM NaHCO_3_). This was supplemented with NaCl to a final concentration of 300 mM and incubated overnight at 65 °C to reverse the crosslinks. The samples were treated with 2 μg of Proteinase K (Roche, Switzerland) for 2 h at 45 °C after addition of 40 mM EDTA and 40 mM Tris-HCl (pH 6.5). DNA was extracted using phenol:chloroform:isoamyl alcohol (25:24:1), ethanol-precipitated using 20 μg of glycogen as carrier, and resuspended in 50 μL of sterile deionized water. The comparative ChIP-followed by deep Sequencing (ChIP-Seq), was done using the next generation sequencing on an Illumina platform at Fasteris, Switzerland.

### 2.6 ChIP-Seq data analysis

The FASTQ files were checked for quality of sequencing using FastQC software, version 0.11.5. The first ten bases showed distortion, due to which it was decided to trim the first ten bases from all short reads. The reads were trimmed at the 5′ end for 10 bases using fastx_trimmer tool from Fastx-toolkit version 0.0.14. The preprocessed reads were mapped to the *Caulobacter crescentus* NA1000 reference genome (CP001340.1) using aligner Bowtie version 1.0.0 using the following parameter: −m 1, −S, −v 2. Around 36.9 million reads mapped uniquely to the reference genome for the wild-type *cckA* and 21 million reads for the mutant *cckA*(L228P) strains.

Further, the aligned reads were imported onto Seqmonk (version 1.38.1) to build the sequence read profiles. The genome was subdivided into 50 bp probes and a value representing the number of reads mapping to the genome within a probe was calculated using the Read Count Quantitation option. The probe list with the quantified value for each probe was exported. Custom Perl scripts were used to compute the relative abundance of each probe as a percent with respect to the total uniquely mapped reads for each dataset. A cutoff was determined as average reads plus twice the standard deviation of the sample to differentiate between candidate peaks and background noise. The candidate peaks were annotated using custom Perl scripts. A probe was annotated with a gene if the centre of the probe was within a distance of −500 and +100 bases from the transcription start site of the gene, taking into account the orientation of the gene as well. If a probe is found to satisfy the condition for two genes (each on either strand), then both genes are reported. Probes without an annotation are labeled as ‘NO ANNO’.

### 2.7 Quantitative PCR (qPCR) analyses

qPCR was performed on a CFX96 Real Time PCR System (Bio-Rad, CA, USA) using 10% of each ChIP sample, 12.5 μL of SYBR® green PCR master mix (Bio-Rad, CA, USA), 200 nM of primers and 6.5 μL of water per reaction. Standard curve generated from the cycle threshold (Ct) value of the serially diluted chromatin input was used to calculate the % input value of each sample. Average values are from triplicate measurements done per culture. The final data was generated from three independent cultures. The Standard Error (SE) shown in the figures was derived with Origin 7.5 software (OriginLab Corporation, Northhampton, MA, USA). C_*ori*__Fwd (5′-CGCGGAACGACCCACAAACT-3′) and C_*ori*__Rev (5′-CAGCCGACCGACCAGAGCCA-3′) primer pairs as described earlier [35] were used to amplify the region near *ori* precipitated by anti-CtrA antibody. To check CtrA binding on P_*pilA*_, the DNA region analyzed by real-time PCR was from nucleotide −287 to −91 relative to the start codon of *pilA* [31]. A P_*kidO*_ fragment comprising nt 3,857,810–3,858,141 of the NA1000 genome sequence was quantified [36] to monitor CtrA binding on P_*kidO*_. To quantify CtrA occupancy at the promoter of *flbT*, the DNA region, −280 to +30 relative to the start codon of *flbT* was used. The DNA region from −226 to +30 relative to the start codon of *tacA* was analyzed for quantifying CtrA occupancy on P_*tacA*_ [31]. The DNA region from −263 to −50 relative to the start codon of *fliQ* was quantified to assess CtrA binding at the promoter of *fliQ*. For monitoring the CtrA occupancy at the promoter of *sciP*, the DNA region −256 to −20 relative to the start codon of *sciP* was used.

### 2.8 Microscopy

Differential interference contrast (DIC) and fluorescence microscopy were performed on a Nikon Eclipse 90i microscope equipped with 100X oil TIRF (1.49 numerical aperture) objective and a CoolSNAP HQ-2 (Photometrics, USA) CCD camera. Cells were placed on 1% agarose pads for imaging. Images were processed and analyzed with the Metamorph software (Molecular Devices, USA).

### 2.9 β‑Galactosidase assay

The cultures harboring the *lacZ* reporter plasmids were incubated at 29 °C till they reached 0.1–0.4 OD@660 nm (A_660_). 50 μL of the cells were treated with 10 μL of chloroform followed by the addition of 750 μL of *Z*-buffer (60 mM Na_2_HPO_4_, 40 mM NaH_2_PO_4_, 10 mM KCl, 1 mM MgSO_4_·7H_2_O, pH 7.0) followed by 200 μL of Ortho Nitro Phenyl‑β‑d‑Galactoside (from stock concentration of 4 mg/mL dissolved in 100 mM potassium phosphate buffer [pH 7.0]). The reaction mixture was incubated at 30 °C till yellow color developed. Finally, 500 μL of 1 M Na_2_CO_3_ solution was added to stop the reaction and absorbance at 420 nm (A_420_) of the supernatant was noted using *Z*-buffer as the blank. The Miller units (U) were calculated using the equation U = (A_420_ × 1000) / (A_660_ × t × v), where ‘t’ is the incubation time (min), ‘v’ is the volume of culture taken (mL). Experimental values were average of three independent experiments. The SE shown in the figures was derived with Origin 7.5 software (OriginLab Corporation, Northhampton, MA, USA).

### 2.10 Immunoblots

Proteins from cell lysates were allowed to migrate on a polyacrylamide gel. Subsequently, the proteins were transferred onto poly vinylidene fluoride membrane (PVDF, Millipore), at the cold room, by applying constant voltage. The blots were blocked for 1 h at room temperature with TBST solution (20 mM Tris-HCl [pH 7.5], 150 mM NaCl, 0.5% Tween-20, 5% nonfat dry milk), followed by 1 h incubation in TBST solution with primary antibodies, anti-CtrA (31) (1:10000), anti-CckA (15) (1:10000), anti-DnaA [5] (1:10000) and anti-MreB [37] (1:30000 fold dilution). The blots were then washed four times with TBS solution (20 mM Tris-HCl [pH 7.5], 150 mM NaCl), and detected with donkey anti-rabbit secondary antibody conjugated to horseradish peroxidise (Jackson ImmunoResearch, PA, USA).

## 3 Results

### 3.1 Suppressor mutations in CckA alleviate NstADD toxicity

Overproduction of the cell-cycle stable form of the Topo IV inhibitor, NstADD, leads to fitness defect and impaired chromosome segregation in *Caulobacter* [26]. To unearth the signaling network that regulates NstA, we exploited the lethality induced by NstADD overproduction in a genetic screen to identify extragenic suppressors that could tolerate NstADD toxicity (see Materials and Methods). Strikingly, whole-genome sequencing revealed that all nine extragenic suppressors harbored a mutation in the gene encoding the cell cycle histidine kinase, *cckA* (Supplementary Fig. S1B). The suppressor mutations corresponded to CckA-(L228P), (A317V), (D364G), (R356C), (F392L), (F493C) and (F496C) (Fig. 1C, Supplementary Figs. S1A, S2A and S2B, Supplementary Table S1). Interestingly, all the suppressor mutations except the L228P substitution were located either in the histidine kinase domain or the ATP binding domain of CckA (Supplementary Fig. S1B). Remarkably, the L228P mutation, which mapped to the PAS-B domain in CckA, rendered developmental defects such as cell filamentation in the wild-type (*WT*) and the ∆*nstA* mutant (Fig. 1D, Supplementary Fig. S2A). To confirm that it is the L228P mutation in *cckA* that conferred resistance to NstADD toxicity, we backcrossed the *cckA*(L228P) mutation into a clean *WT* or a ∆*nstA* strain by transduction (see materials and methods). The backcrossed cells were indeed able to tolerate the NstADD overexpression (Fig. 1C). The fact that the CckA(L228P) mutation was not in the kinase or the ATP binding domain of CckA, prompted us to investigate further the mechanism by which the *cckA*(L228P) mutant induced the developmental defects and negated the cell viability defect attributed by NstADD in *Caulobacter*.

### 3.2 CckA(L228P) mutation leads to elevation of CtrA~P levels

**Fig. 2:**
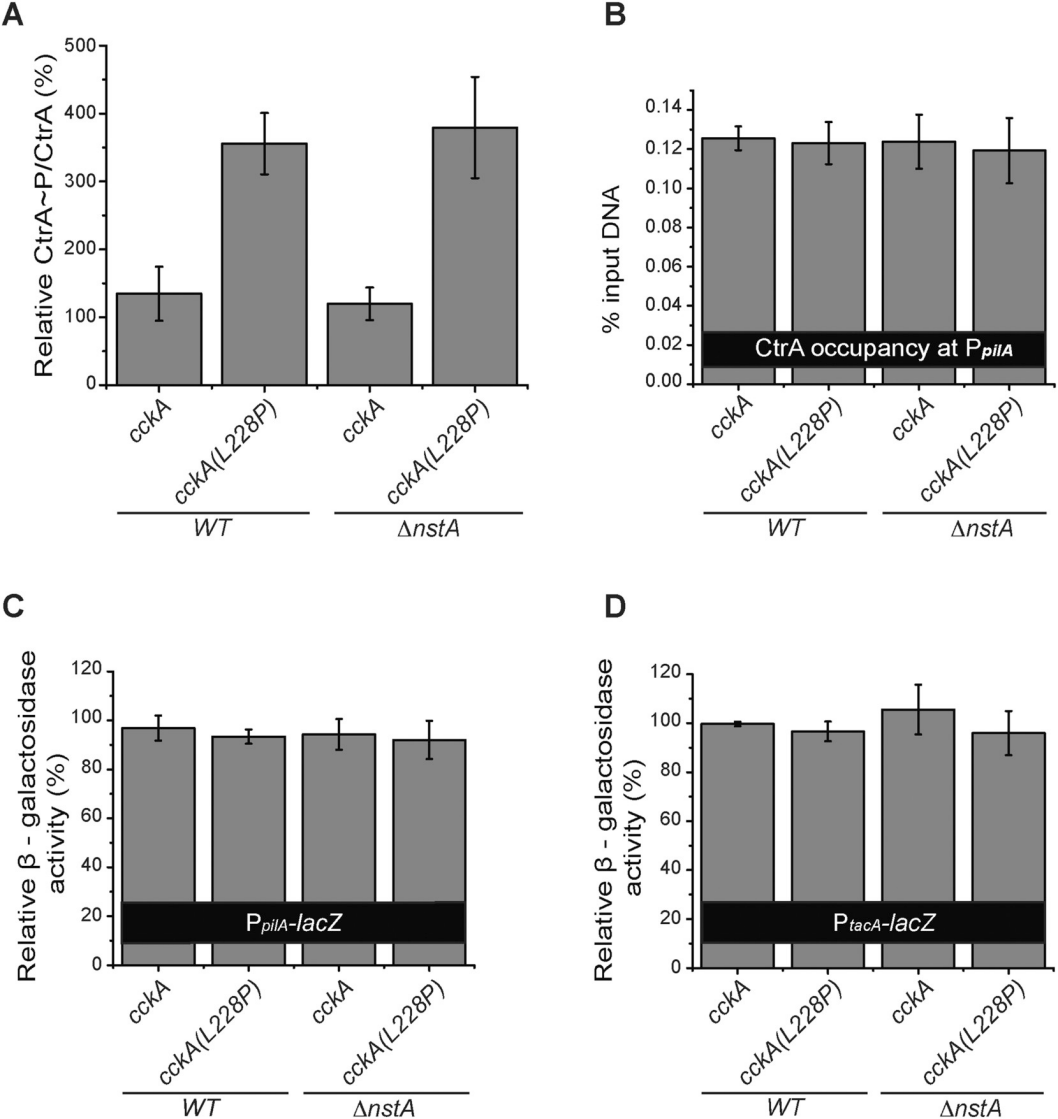
Effect of CckA(L228P) mutation on CtrA. (A) *In vivo* phosphorylation experiment denoting CtrA~P/CtrA levels in *WT*, *WT cckA*(L228P), ∆*nstA* and ∆*nstA cckA*(L228P) mutants. (B) Data of qChIP analysis showing the CtrA occupancy at the promoter region of *pilA* (P_*pilA*_) in *WT*, *WT cckA*(L228P), ∆*nstA* and ∆*nstA cckA*(L228P) cells. The relative β‑galactosidase activity (in percentage) of (C) the P_*pilA*_-*lacZ* and (D) the P_*tacA*_-*lacZ* reporters in *WT*, *WT cckA*(L228P), ∆*nstA* and ∆*nstA cckA*(L228P) cells. The values ± SE, represented in A, B, C and D are the average of at least three independent experiments.

The cell cycle histidine kinase, CckA, is the primary kinase that activates the master cell cycle transcriptional regulator, CtrA, by phosphorylation [11]. The phosphorylated form of CtrA (CtrA~P) binds efficiently to its target promoters on the chromosome, regulating transcription [11,12,38]. Therefore, we decided to investigate if the L228P mutation in the PAS-B domain of CckA could affect CtrA~P levels. Interestingly, *in vivo* phosphorylation analysis revealed that the relative levels of CtrA~P, compared to total CtrA, were approximately three-fold higher in the *WT* or the ∆*nstA* cells harboring the *cckA*(L228P) mutation (Fig. 2A). The total CtrA levels remained unaltered in the *WT cckA*(L228P) and the ∆*nstA cckA*(L228P) mutants when compared to *WT* or ∆*nstA* cells that did not carry the mutation in *cckA* (Supplementary Fig. S3A). To analyze if this increase in CtrA~P levels is due to an increase in the kinase activity of CckA, we measured auto-phosphorylation levels of CckA (CckA~P) by *in vivo* phosphorylation in ∆*nstA* and ∆*nstA cckA*(L228P) genetic backgrounds. The relative levels of CckA~P, to total CckA, was indeed found to be at least two-fold higher, than ∆*nstA*, in the ∆*nstA* cells harboring the *cckA*(L228P) mutation (Supplementary Fig. S3B), while the total CckA protein levels were not significantly altered (Supplementary Fig. S3C).

It has been shown that an increase in CtrA~P levels could result in elevated levels of CtrA binding to its target promoters [34,39]. Therefore, we decided to analyze the binding of CtrA~P, and its effect, on well-established CtrA-dependent promoters such as *pilA*, *tacA*, *sciP* and *kidO* [31,36,40], by quantitative chromatin immunoprecipitation (qChIP) analysis using CtrA specific antibodies and β‑galactosidase (LacZ)-based promoter-probe assays. Surprisingly, our qChIP experiments revealed that the binding of CtrA to the P_*pilA*_, P_*kidO*_, P_*tacA*_ and P_*sciP*_ promoters were not significantly different in the *cckA*(L228P) mutants when compared to *WT* or ∆*nstA* (Fig. 2B, Supplementary Fig. S3D–F). Further, our LacZ-based promoter-probe assays using the promoters of *pilA* (P_*pilA*_-*lacZ*) and *tacA* (P_*tacA*_-*lacZ*) in *WT*, ∆*nstA*, *WT cckA*(L228P), and ∆*nstA cckA*(L228P) genetic backgrounds, revealed that there were no measurable differences in the P_*pilA*_ and P_*tacA*_ promoter activities in the *cckA*(L228P) mutants (Fig. 2C, D).

Collectively, these results indicated that the *cckA*(L228P) mutation increased CtrA~P levels by enhancing the CckA kinase activity. Nevertheless, this surge in CtrA~P levels did not result in increased binding of CtrA, or elevated promoter activity, at least in the G1-specific promoters of CtrA such as P_*kidO*_, P_*pilA*_ and P_*tacA*_.

### 3.3 CckA influences the promoter specific binding of CtrA

**Fig. 3:**
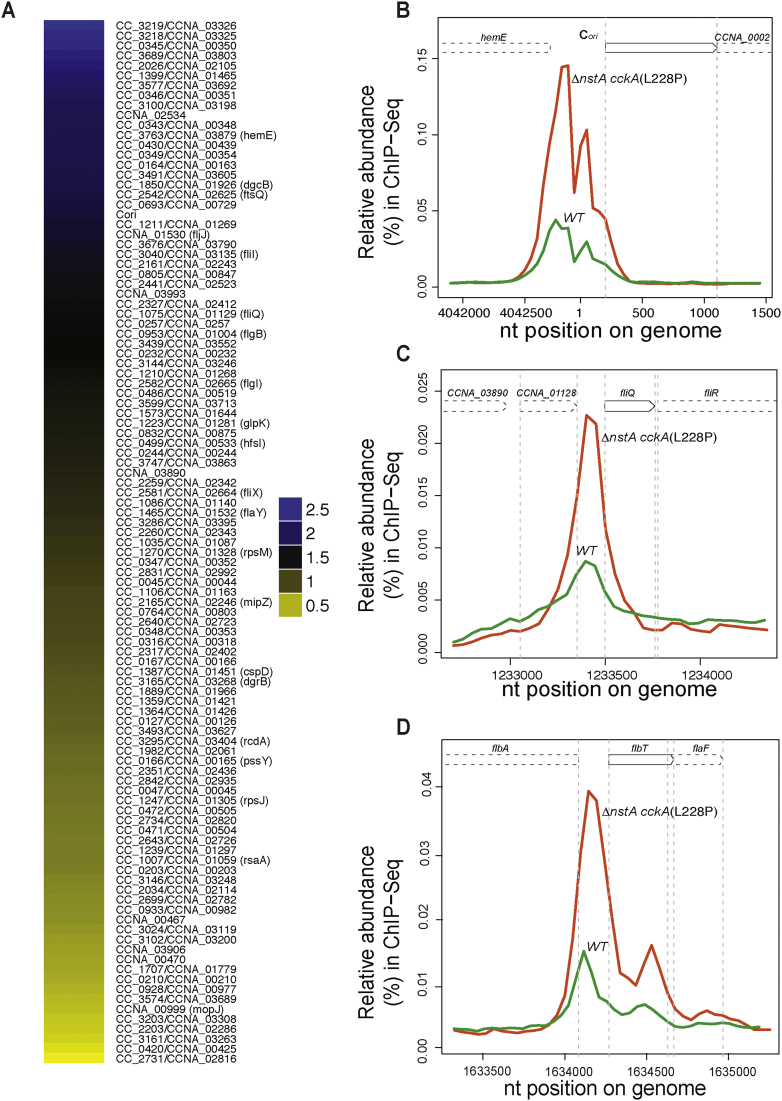
The CckA(L228P) mutation leads to differential DNA binding of CtrA. (A) Genome-wide comparative ChIP-Seq using polyclonal antibodies to CtrA, denoting the occupancy of CtrA on the chromatin of *WTcckA vs* ∆*nstA cckA*(L228P) mutant cells. The color key (log_2_ ratio) indicates the degree by which the occupancy of CtrA is varied, at the promoters of selected target genes, as a result of the CckA(L228P) substitution (see Supplementary Dataset 1 for the complete list of targets). Traces of the occupancy of CtrA at (B) the chromosomal origin of replication, C_*ori*_, (C) the promoter of *fliQ* and (D) the promoter of *flbT*. Fig. 3B–D were derived from the ChIP-Seq data and the traces of CtrA in the *WT* is denoted in green, and in the ∆*nstA cckA*(L228P) mutant is denoted in red. Also see Supplementary Fig. S4.

Next, we decided to investigate if the unchanged DNA binding of CtrA, despite increased CtrA~P levels in the *cckA*(L228P) mutant, is specific to a subset of CtrA dependent promoters. Towards this, we performed chromatin immunoprecipitation followed by deep sequencing (ChIP-Seq) to analyze the CtrA occupancy on its target promoters in the *cckA* and *cckA*(L228P) mutant backgrounds on a genome-wide scale. From the ChIP-Seq analyses it was evident that the *cckA*(L228P) mutation enhanced the CtrA occupancy at the promoter regions of target genes whose transcripts peaked at S-phase, including the Class II flagellar genes such as *pleA*, *fliQ*, *fliL*, *fliM*, *fliJ* and *fliI* [41], pilus secretion genes, *cpaA* and *cpaB* [42], flagellar regulatory genes, *flbT* and *flbA* [43], and the chemotaxis genes, *motA* and *motB* [44] (Fig. 3A, C, D and Supplementary Dataset 1). Further, the qChIP experiments confirmed the enhanced binding of CtrA at the promoter regions of *fliQ* and *flbT* in the *WT* *cckA*(L228P) and ∆*nstA cckA*(L228P) genetic backgrounds when compared to *WT* or ∆*nstA* (Supplementary Fig. S4A, B). To corroborate if this increase in binding of CtrA to these promoters resulted in an increased promoter activity, we analyzed the activity of the *fliM* promoter (P_*fliM*_) and the *flbT* promoter (P_*flbT*_) using LacZ-reporter-based fusions to these promoters (P_*fliM*_-*lacZ* and P_*flbT*_-*lacZ*). Our analyses showed that indeed the activities of P_*fliM*_ and P_*flbT*_ were increased in the *cckA*(L228P) mutant background commensurate with the increase in binding of CtrA to these promoters (Supplementary Fig. S5A, B). Further, to analyze if the increase in S-phase specific promoter activity is due to an extended S-phase during the cell cycle, we performed flow cytometry assay to analyze the DNA content in *WT*, ∆*nstA*, *WT cckA*(L228P) and ∆*nstA cckA*(L228P) cells. The flow cytometry analyses revealed that *WT cckA*(L228P) and ∆*nstA cckA*(L228P) mutants did not show significant increase in population of cells in S-phase or undergoing replication when compared to *WT* or ∆*nstA* (Supplementary Fig. S5C), indicating that the increase in activity of S-phase specific CtrA-dependent promoters in the *cckA*(L228P) mutant may not be due to a prolonged S-phase.

Interestingly, in addition to the S-phase specific promoters, the ChIP-Seq data also revealed a significant increase in binding of CtrA to the chromosomal origin of replication, C_*ori*_ (Fig. 3B). Our qChIP experiment also confirmed the increase in CtrA occupancy at the C_*ori*_, in the *cckA*(L228P) mutant. In comparison to the *WT or* ∆*nstA*, a three-fold increase in the binding of CtrA at C_*ori*_ was evident in the *cckA*(L228P) mutant backgrounds (Supplementary Fig. S4C). Together, these data pointed towards the differential DNA binding of CtrA, as a result of the *cckA*(L228P) mutation, to S-phase specific target promoters and C_*ori*_ possibly regulated through the PAS-B domain of CckA.

### 3.4 CtrA mediated repression of replication initiation mitigate NstA induced lethality

**Fig. 4:**
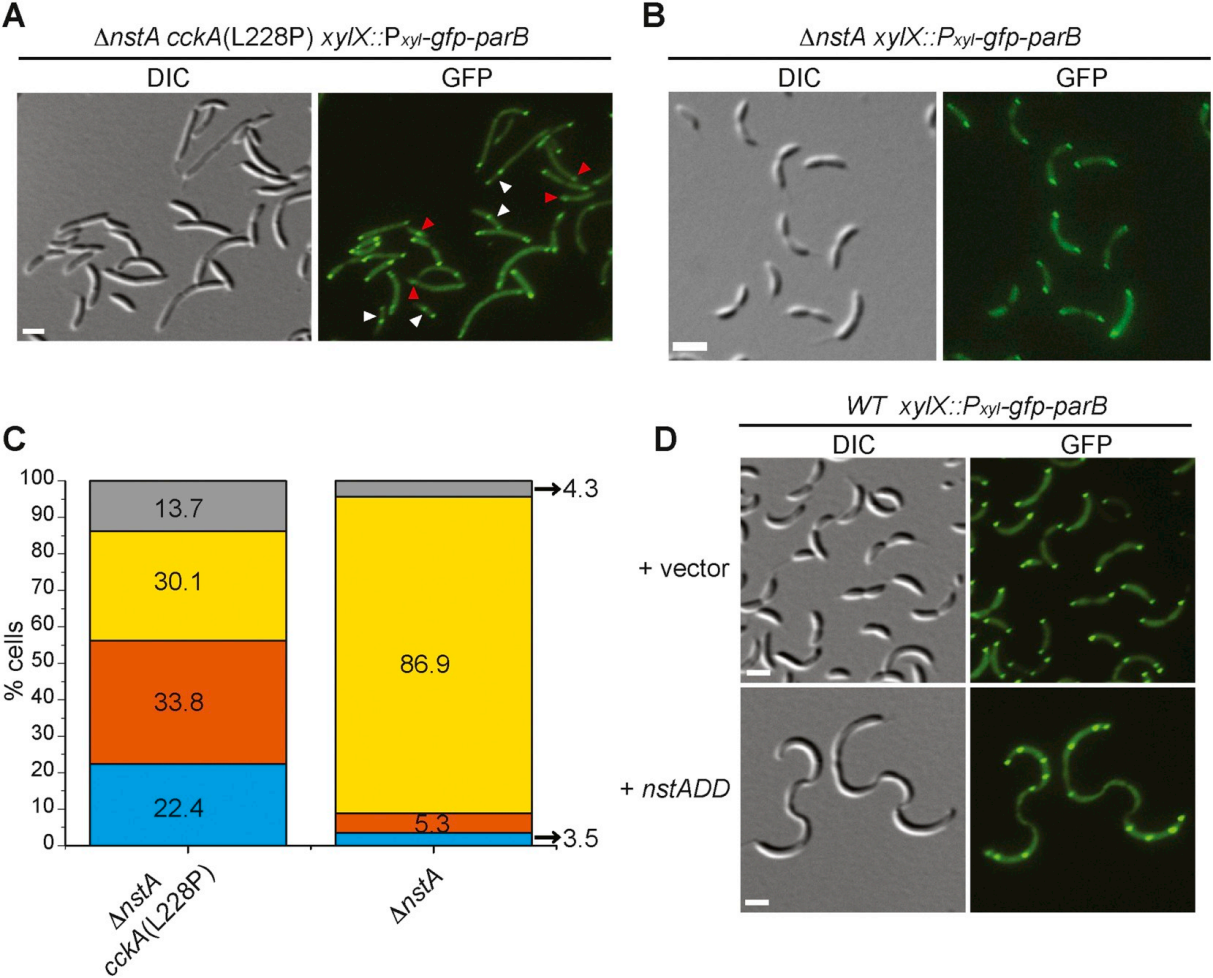
Suppression of NstADD toxicity by the *cckA*(L228P) mutant. (A) DIC and fluorescent micrographs of the extragenic suppressor mutant, ∆*nstA cckA*(L228P), harboring *gfp*-*parB* at the chromosomal *xylX* locus (*xylX::*P_*xyl*_-*gfp*-*parB*). Red arrow-heads: cells with one GFP-ParB foci; white arrow-heads: cells with two partially segregated GFP-ParB foci. (B) DIC and fluorescent micrographs of ∆*nstA* cells expressing *gfp*-*parB* from *xylX::*P_*xyl*_-*gfp*-*parB*. (C) Data representing the stalked cells with one (blue), two partially segregated (orange), normal bipolar (yellow), and multiple (grey) GFP-ParB foci in ∆*nstA cckA*(L228P) (data from 1032 stalked cells) or ∆*nstA* (data from 944 stalked cells). (D) DIC and fluorescent micrographs of *WT* cells harboring *xylX::*P_*xyl*_-*gfp*-*parB*, and overexpressing NstADD from the vanillate inducible promoter (P_*van*_) on pBVMCS-4 or carrying the vector alone. The cells in (A), (B) and (D) were treated with 0.3% xylose to induce the production of GFP-ParB. Cells in (D) were additionally treated with 0.5 mM vanillate for 3 h to induce NstADD production. Scale bar in A, B and D: 2 μm.

**Fig. 5:**
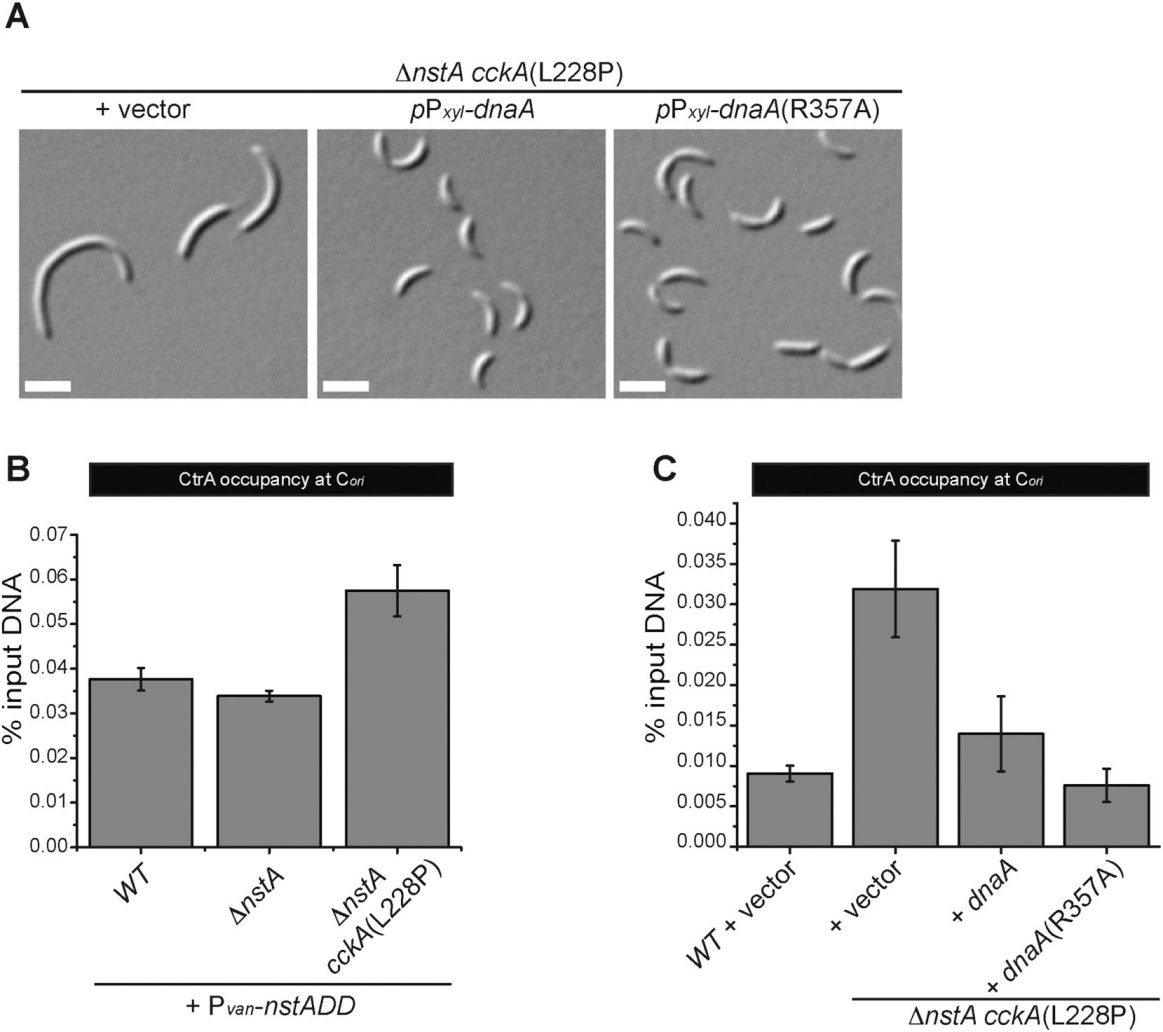
DnaA overexpression can alleviate the filamentation phenotype induced by the CckA(L228P) mutation. (A) DIC images showing ∆*nstA cckA*(L228P) cells harboring the vector or expressing *dnaA* or *dnaA*(R357A) from the xylose inducible promoter (P_*xyl*_) on the medium copy vector, pJS14. The cells were grown to exponential phase in PYE supplemented with 0.2% glucose, prior to the addition of 0.3% xylose. The xylose induction was done for 6 h. (B) qChIP data depicting the CtrA occupancy at C_*ori*_ in *WT*, ∆*nstA*, and ∆*nstA cckA*(L228P) genetic backgrounds, after the overexpression of NstADD from the vanillate inducible promoter (P_*van*_) on pBVMCS-4. The cells were treated with 0.5 mM vanillate for 3 h to induce the production of NstADD. (C) The qChIP data showing the CtrA occupancy at C_*ori*_ in ∆*nstA cckA*(L228P) cells, after the overexpression of DnaA or DnaA(R357A) from pJS14. The cells were treated in the same manner, as described in (A). The values ± SE, represented in (B) and (C) are the average of at least three independent experiments.

Next, we wondered if the increase in the CtrA binding to the C_*ori*_ is what contributes to the rescue of the toxicity induced by the overproduction of stable NstADD. We hypothesized that the specific modulation of CtrA activity by the CckA(L228P) mutation leading to enhanced binding of CtrA to the C_*ori*_ may be delaying replication initiation. Further, this delay in replication initiation may be slowing down the chromosome cycle to compensate for the chromosome segregation defect caused by the reduction in the decatenation activity of Topo IV by NstADD [26]. To test this hypothesis, we decided to monitor the appearance or movement of the newly replicated chromosomal origin in the *cckA*(L228P) mutant. In *Caulobacter*, it has been well demonstrated that the newly formed origin of replication is immediately tethered to the opposite pole upon initiation of replication [45]. The chromosome partitioning protein, ParB, specifically binds to the regions near C_*ori*_ and moves along with the C_*ori*_ upon initiation of replication [46,47]. Therefore, the movement of fluorescently tagged ParB, GFP-ParB, can be used as a proxy to monitor the movement of the newly formed C_*ori*_ [47,48]. Localization experiments using GFP-ParB showed that the ∆*nstA cckA*(L228P) mutant had stalked cells with either single GFP-ParB foci (22.4%; Fig. 4A red arrow heads, Fig. 4C), or with two GFP-ParB foci, partially segregated, with the second foci still migrating to the opposite pole (33.8%; Fig. 4A white arrowheads, Fig. 4C). This was unlike in ∆*nstA* cells wherein the newly replicated C_*ori*_ along with GFP-ParB was immediately tethered to the opposite pole upon initiation of replication (86.9%; Fig. 4B and C). From this observation, we inferred that in the *cckA*(L228P) mutant, the initiation of replication and the elongation processes of the chromosome may be slow. The *C*_*ori*_ to *ter* ratios in the *WT cckA*(L228P) or ∆*nstA cckA*(L228P) strains further indicated that the *cckA*(L228P) mutation may be affecting the initiation of chromosome replication (Supplementary Fig. S6A). This slow down may well be due to the increase in CtrA binding to the C_*ori*_. We also observed multiple GFP-ParB foci in *Caulobacter* cells, overproducing NstADD (Fig. 4D), unlike the control samples with pBVMCS-4 vector alone, wherein, bipolar GFP-ParB foci were predominant (Fig. 4D). Thus we surmise that in the cells overproducing NstADD, multiple rounds of DNA replication are initiated and the chromosome decatenation is hampered [26]. To counter this effect, *cckA*(L228P) point mutation enhances the CtrA binding at the C_*ori*_ (Fig. 3B, Supplementary Fig. S4C), which can significantly slow down the replication cycle. Our hypothesis was further corroborated by the observation that the increase in the binding of CtrA to the C_*ori*_ is still retained after the overexpression of NstADD. In comparison to the *WT* or ∆*nstA*, overexpressing NstADD, the *cckA*(L228P) mutant cells overproducing NstADD, had a significant increase in the occupancy of CtrA at the C_*ori*_ (Fig. 5B). To analyze if this effect on replication initiation is due to a disturbance in the production of the Topo IV subunits, ParC or ParE, we analyzed the promoter activity of *parC* (P_*parC*_) and parE (P_*parE*_) using LacZ-based reporters (P_*parC*_-*lacZ* and P_*parE*_-*lacZ*). The P_*parC*_-*lacZ* and P_*parE*_-*lacZ* activity assays indicated that the *cckA*(L228P) mutation did not alter the production of *parC* or *parE* in the *WT* or the ∆*nstA* cells (Supplementary Fig S6B, C). This observation suggested that the *cckA*(L228P) mutation enhanced the CtrA occupancy at the C_*ori*_ to slow down the replication cycle without affecting the transcription of the Topo IV encoding genes.

The CtrA binding boxes at the origin overlap with the DnaA binding sites [7,49]. Thus when CtrA~P is abundant in the system, the DnaA binding to the origin is inhibited leading to inhibition of chromosome replication initiation [50]. Therefore, we speculated that, if it is the increase in CtrA binding that is leading to slow down in replication, then such an inhibition should be relieved by titrating out CtrA at the C_*ori*_ by the overexpression of DnaA. Indeed, the overproduction of DnaA or its constitutively active ATP bound form, DnaA(R357A), from the xylose inducible promoter (P_*xyl*_) on a medium copy plasmid [35] caused a considerable decrease in cell filamentation of ∆*nstA cckA*(L228P) cells (Fig. 5A). In addition, qChIP experiments using CtrA specific antibodies also confirmed that the CtrA occupancy at C_*ori*_ was greatly reduced, by about 70%, post the DnaA/DnaA(R357A) overexpression in the Δ*nstA cckA*(L228P) mutant (Fig. 5C). Further, immunoblots using DnaA antisera [5] revealed that the *cckA*(L228P) mutation did not affect DnaA abundance suggesting that the increase in CtrA occupancy at C_*ori*_ is not due to a decrease in availability of DnaA (Supplementary Fig. S3A). Altogether, these results indicated that the increased binding of CtrA specifically to the C_*ori*_, facilitated by the CckA(L228P) mutation, slows down the DNA replication cycle to alleviate the toxicity attributed to NstADD overproduction.

## 4 Discussion

The highly conserved CckA-CtrA signal transduction pathway in α-proteobacteria has several implications in development and pathogenesis. For example, during the early stages of symbiosis, in the nitrogen fixing bacteria, *Sinorhizobium meliloti*, the role of CckA and its regulation has been shown to be essential [51]. Likewise, the viability of the intracellular pathogen, *Brucella abortus*, in the human macrophages is dependent on the CckA-ChpT-CtrA pathway [52]. The regulatory networks involving CtrA can be related to the specific lifestyle of the bacterium. For instance, while in *Caulobacter* CtrA is involved in cell-fate control, and cell cycle, by fine-tuning the stalked and swarmer cell programs, the control of cell envelope composition by CtrA is crucial in *B*. *abortus* and *Rhizobium leguminosarum* [53,54], thereby reiterating the plasticity of CckA-CtrA pathway.

**Fig. 6:**
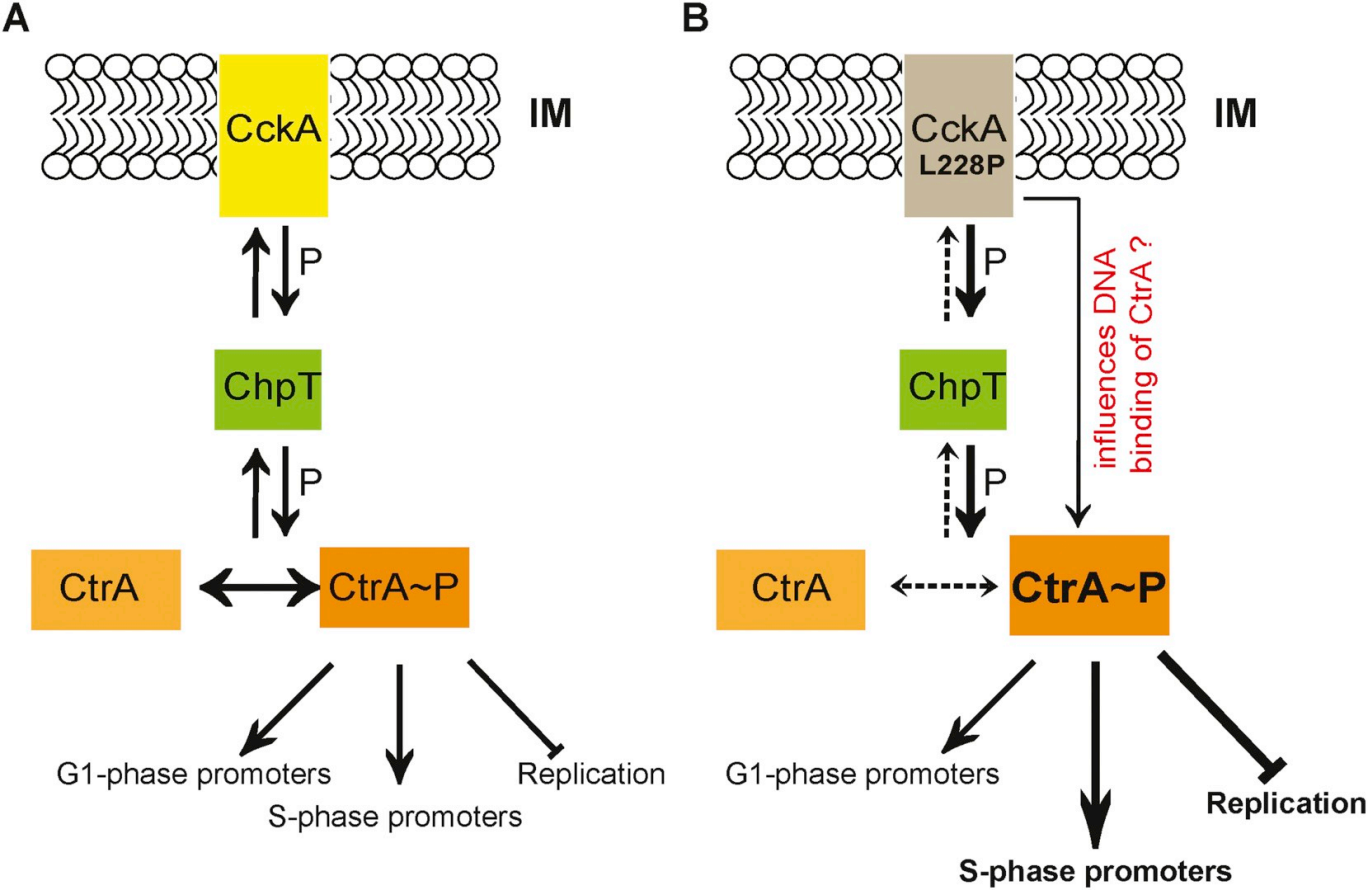
Model for CckA(L228P) function. (A) The membrane bound bi-functional kinase/phosphatase CckA (yellow), in its kinase form, phosphorylates CtrA (orange), *via* the intermediate phosphotransferase, ChpT (green). The active phosphorylated form of CtrA (CtrA~P) binds to DNA and triggers the transcription of G1- and S-phase specific CtrA-dependent promoters during the cell cycle. CtrA~P also inhibits the initiation of chromosome replication in the G1 cells by binding to the C_*ori*._ When the phosphatase activity of CckA is predominant (see Fig. 1A), the phosphate flow reverses dephosphorylating and inactivating CtrA. (B) The CckA(L228P) mutation leads to a predominant CckA kinase activity thereby increasing CtrA~P levels. In addition, the CckA(L228P) mutation, through an yet to be understood mechanism, specifically increases the CtrA~P binding at C_*ori*_ and at certain CtrA-dependent S-phase specific promoters resulting in an enhanced inhibtion of chromosome replication, and an enhanced activation of a subset of S-phase specific promoters respectively.

In this study, we show that the L228P mutation in the PAS-B domain of CckA not only increases the CtrA~P levels but also rewires the preferential binding of CtrA to its target promoters (Figs. 2A, 3 and Supplementary Dataset 1). The PAS-B domain in CckA has been shown to be necessary for the regulation of its auto kinase activity, and for the switching of CckA between the kinase and the phosphatase modes [23]. The CckA phosphatase activity during the swarmer to stalked cell transition is triggered by the binding of the effector molecule, *c*-di-GMP, to the PAS-B domain [[23], [24], [25]]. Therefore, it is conceivable that the *cckA*(L228P) mutation possibly perturbs the binding of *c*-di-GMP to CckA thereby locking CckA in a kinase active form leading to increased CtrA~P levels in the *cckA*(L228P) mutant (Fig. 6).

Surprisingly, the above-mentioned increase in the CtrA~P levels does not translate into a uniform increase in the binding of CtrA on all its target sites on the chromosome. The increased binding happens only at the C_*ori*_ and a sub-set of S-phase specific CtrA-dependent promoters. Previous studies have shed light on the additional components, SciP and MucR, which modulate the activity of CtrA-dependent promoters during the cell cycle [[55], [56], [57]]. While MucR specifically represses G1-phase promoters of CtrA, SciP has been shown to negatively regulate the CtrA-dependent promoters whose activity are known to peak at the S-phase of the cell cycle [56]. Interestingly, our comparative ChIP-Seq analysis revealed that the CckA(L228P) substitution contributes to the specific enhancement of CtrA binding at the S-phase promoters, which are also bound by SciP. The mechanistic mode of repressing the CtrA transcription by SciP involves a direct interaction between SciP and CtrA [57]. Intriguingly, SciP does not perturb the DNA binding activity of CtrA, instead it blocks the RNA polymerase recruitment to the CtrA activated promoters [57]. Moreover, SciP itself is under the direct transcriptional control of CtrA [55]. Nevertheless, our qChIP experiments show that the *cckA*(L228P) mutation does not alter the binding of CtrA to the *sciP* promoter (Supplementary Fig. S3F) indicating that the *sciP* transcription may not be perturbed due to the *cckA*(L228P) mutation. Therefore, it is tempting to speculate that the CckA(L228P) substitution possibly facilitates CtrA to overcome the inhibition imparted by SciP either by directly acting on SciP or by making CtrA more potent to compete for the RNA polymerase. It may also be possible that the CckA kinase could be regulating the interaction between SciP and CtrA, in a direct or indirect manner. However, this hypothesis remains to be investigated further and our results pave way for exploring this intriguing aspect of the CckA-CtrA pathway.

## Data deposition

The ChIP-Seq data are deposited in the GEO database under accession number GSE113724.

## Author contributions

S.N. and S.K.R. conceived and designed the research. S.N. and L.K. performed research. S.N., L.K. and S.K.R. analyzed data. S.N. and S.K.R. wrote and revised the manuscript.

## Transparency document

Transparency document. Image 3

## Appendix A Supplementary Information

Supplementary information Image 2

Supplementary Dataset 1 Image 1
