# Supplementary material for: Sensory domain of the cell cycle kinase CckA regulates the differential DNA binding of the master regulator CtrA in Caulobacter crescentus
Source: Biochim Biophys Acta Gene Regul Mech. 2018 Oct;1861(10):952–61. doi: 10.1016/j.bbagrm.2018.08.006 (PMC6169604; doi:10.1016/j.bbagrm.2018.08.006)
Supplement: Supplementary file 2 — Supplementary information [file mmc2.pdf]

Sensory domain of the cell cycle kinase CckA regulates the differential DNA binding of the master regulator CtrA in *Caulobacter crescentus*

SUPPLEMENTARY INFORMATION

A

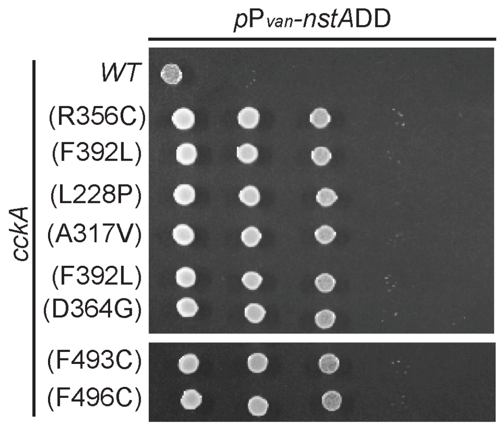

B

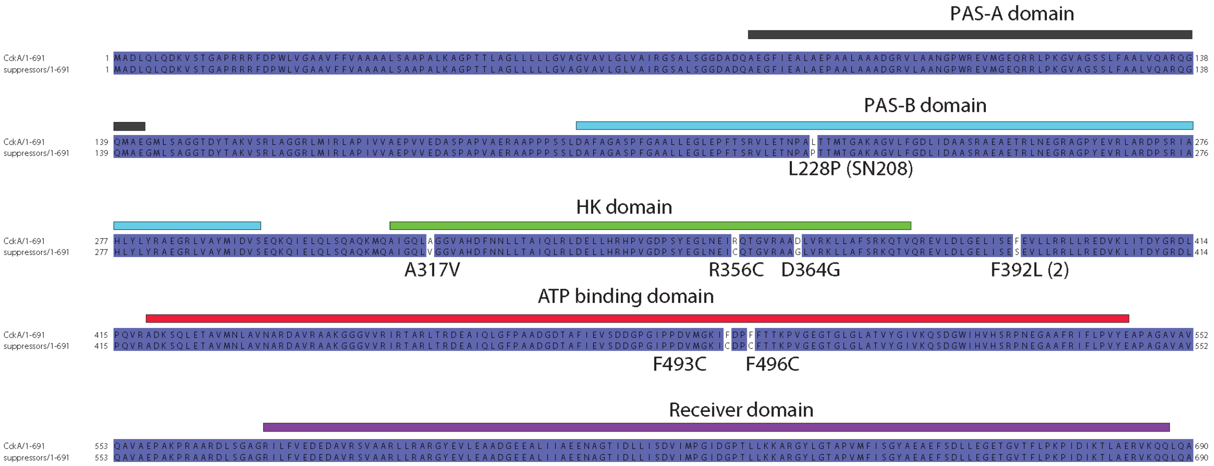

Supplementary Figure S1. (A) Growth of *Caulobacter* harboring the *wild-type cckA*, or NstADD toxicity suppressor mutants of *cckA,* upon *nst*ADD overexpression. The cells were diluted five-fold and spotted on media containing 0.5mM vanillate. The mutant *cckA*(F496C) is in the Δ*nstA* genetic background, rest are in the *WT* background. The only copy of *cckA* on the chromosome is mutated. (B) Schematic denoting the CckA suppressor mutations. The CckA(L228P) mutation resides in the PAS-B domain, whereas the other mutations are harbored either in the histidine kinase domain or the ATP binding domain of CckA.

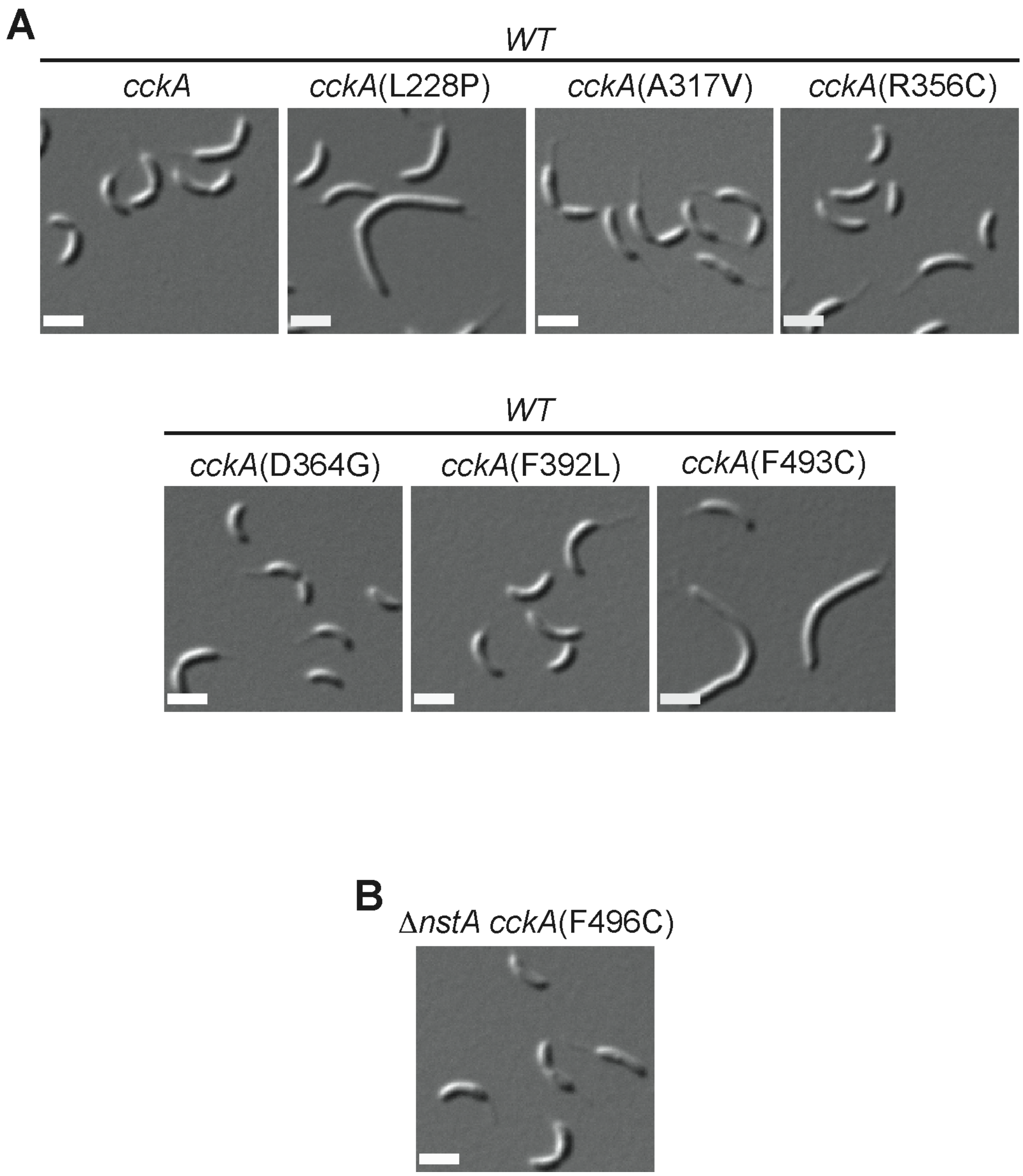

Supplementary Figure S2. DIC images of the plasmid-cured suppressor mutants of *cckA* that alleviate the NstADD overexpression toxicity, in (A) wild-type (*WT*) or (B) Δ*nstA* backgrounds. In both (A) and (B), the only copy of *cckA* on the chromosome is mutated.

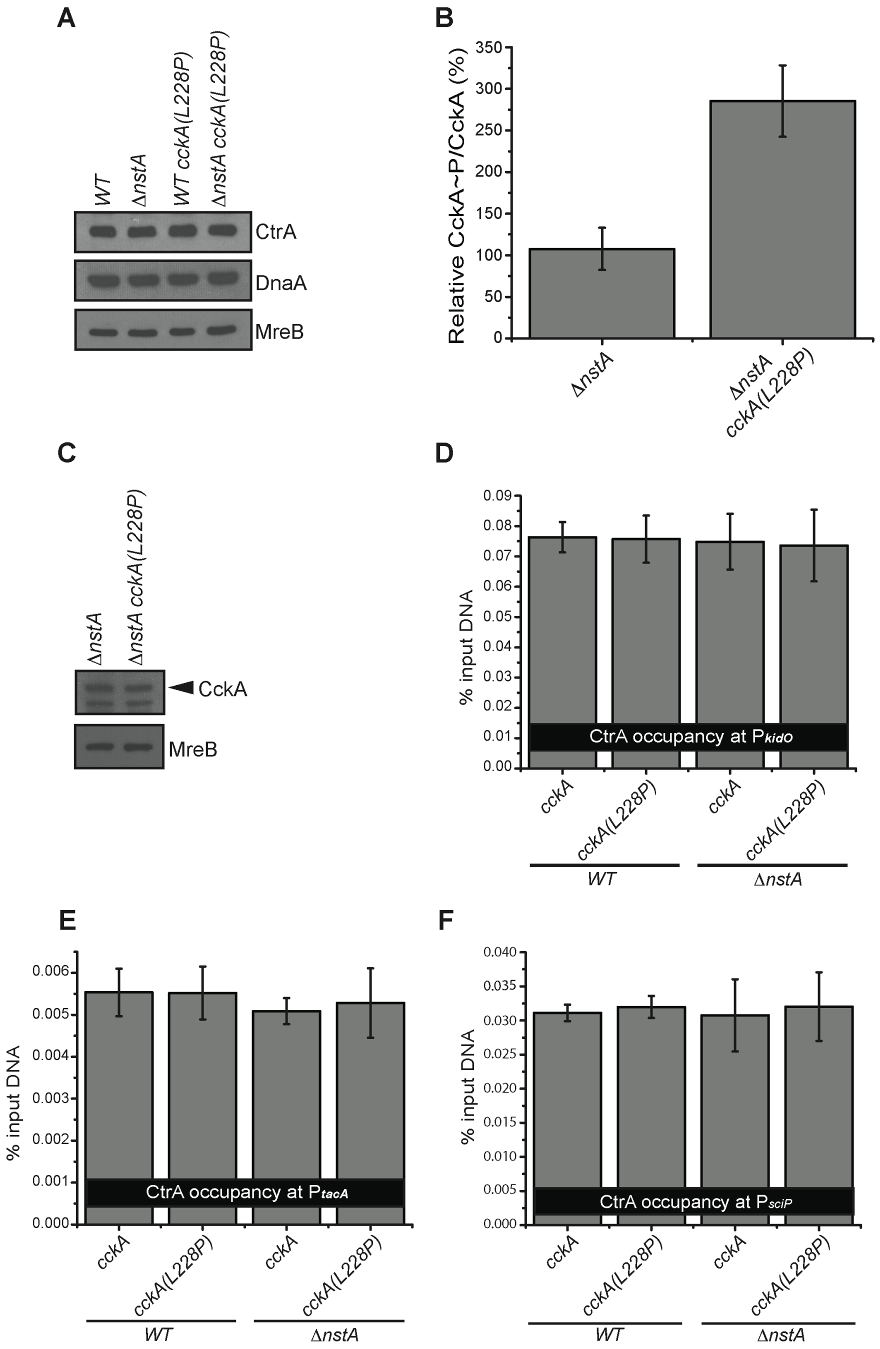

Supplementary Figure S3. (A) Immunoblots showing the steady state levels of CtrA and DnaA in *WT*, Δ*nstA*, *WT cckA*(L228P) and Δ*nstA cckA*(L228P) genetic backgrounds. MreB serves as the loading control. (B) *In vivo* phosphorylation experiment depicting the CckA~P/CckA levels in Δ*nstA* and Δ*nstA cckA*(L228P) mutants. (C) Immunoblots showing the total CckA and MreB levels in the cell lysates from Δ*nstA* and Δ*nstA cckA*(L228P) mutants used in (B). The qChIP data indicating the CtrA occupancy at (D) the promoter of *kidO* (P_{kidO}), (E) the promoter of *tacA* (P_{tacA}), and (F) the promoter of *sciP* (P_{sciP}) in *WT, WT cckA*(L228P), Δ*nstA*, and Δ*nstA cckA*(L228P) cells. The data represented in (B), (D), (E) and (F) are the average of three independent experiments, ±SE.

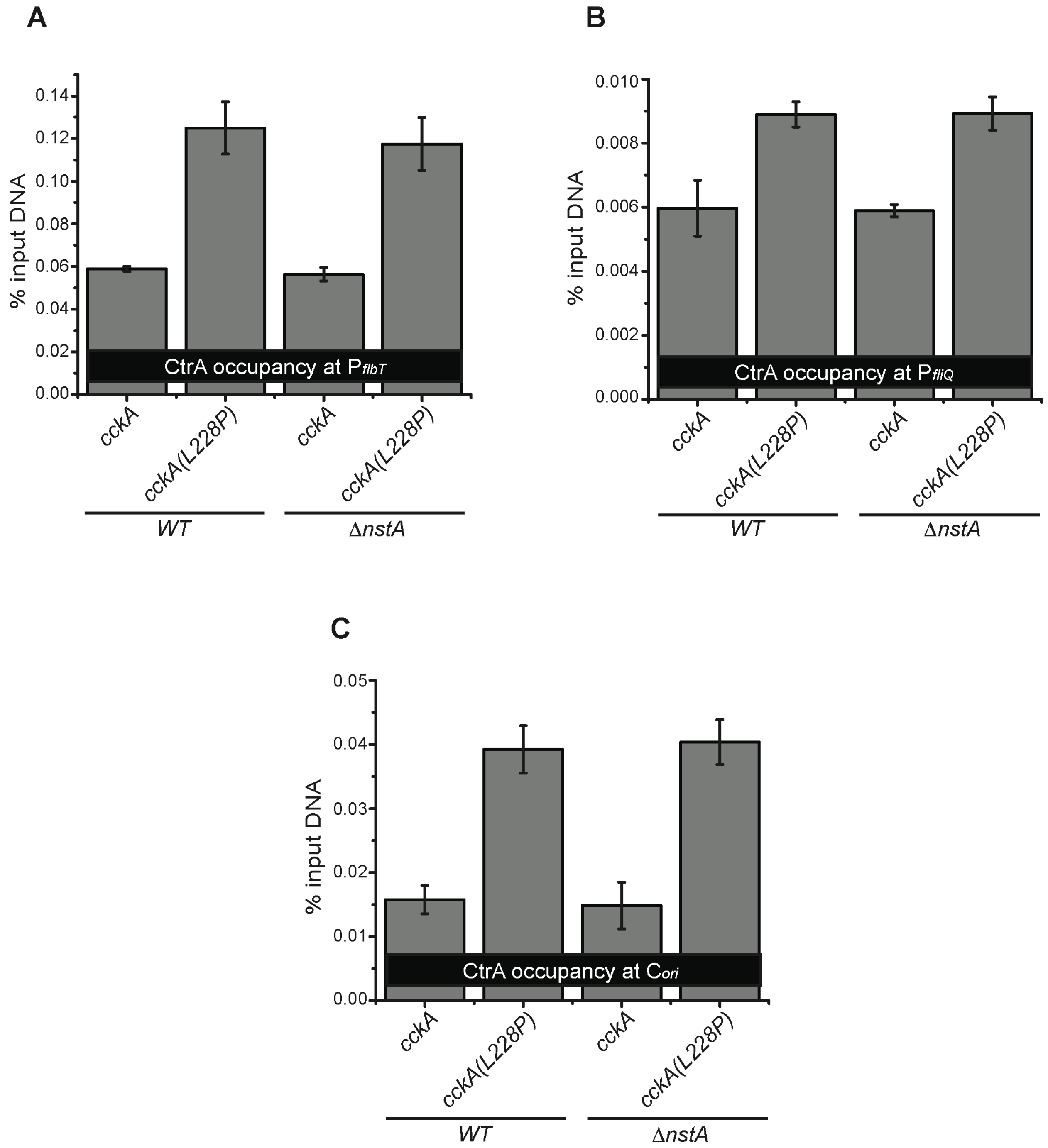

Supplementary Figure S4. qChIP data indicating the CtrA occupancy at (A) the promoter of *flbT* (P$_{flbT}$), (B) the promoter of *fliQ* (P$_{fliQ}$), and (C) at the chromosomal origin of replication, C$_{ori}$ in *WT, WT cckA*(L228P), Δ*nstA*, and Δ*nstA cckA*(L228P) cells. The data represented are the average of three independent experiments, ±SE.

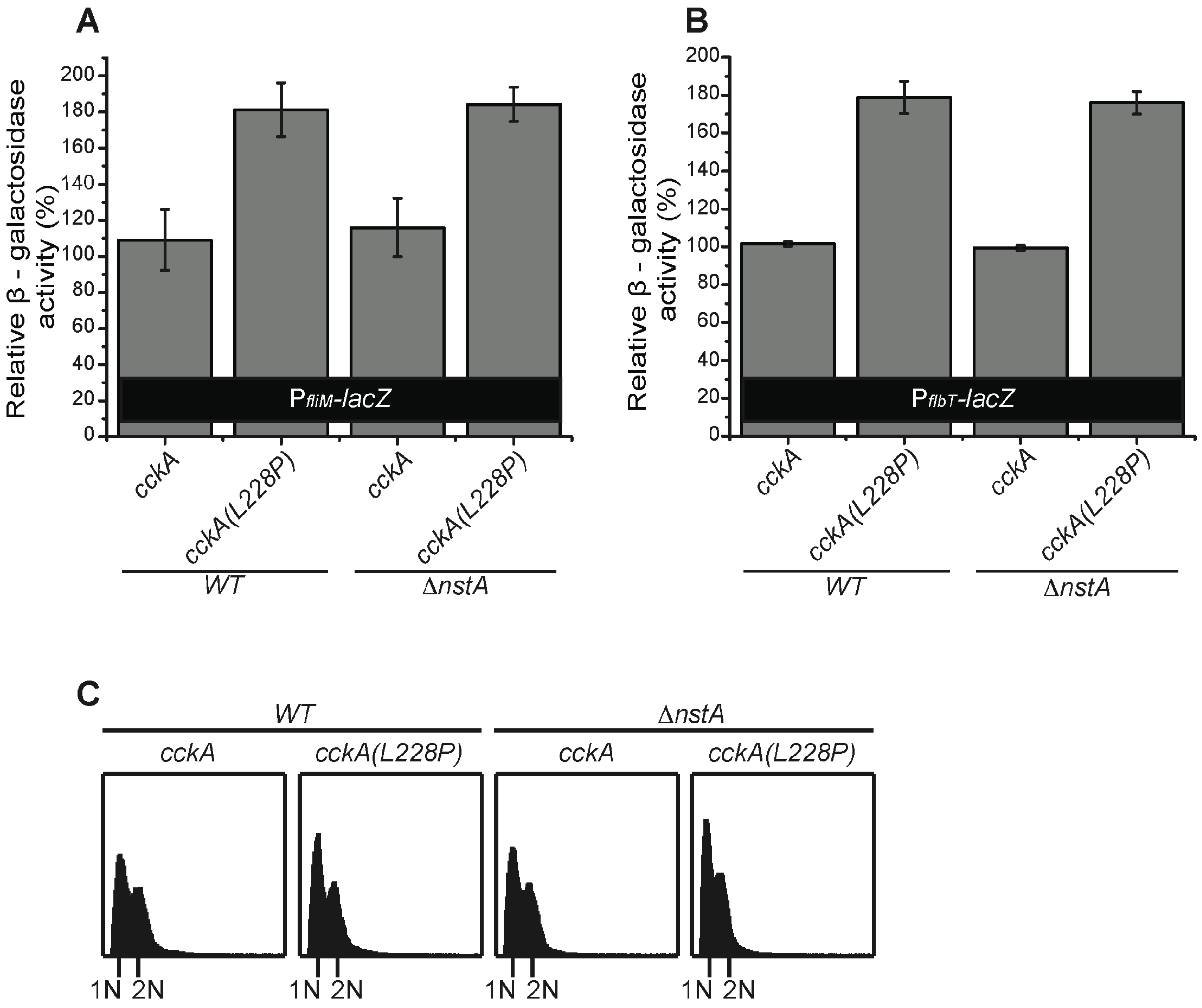

Supplementary Figure S5. Relative β-galactosidase activities of (A) P_{fliM}-*lacZ* reporter, and (B) P_{flbT}-*lacZ* reporter, in *WT, WT cckA*(L228P), Δ*nstA*, and Δ*nstA cckA*(L228P) cells. The values, ±SE, in (A) and (B) are the average of three independent experiments. (C) Flow cytometry profiles showing the DNA content in *WT, WT cckA*(L228P), Δ*nstA*, and Δ*nstA cckA*(L228P) cells.

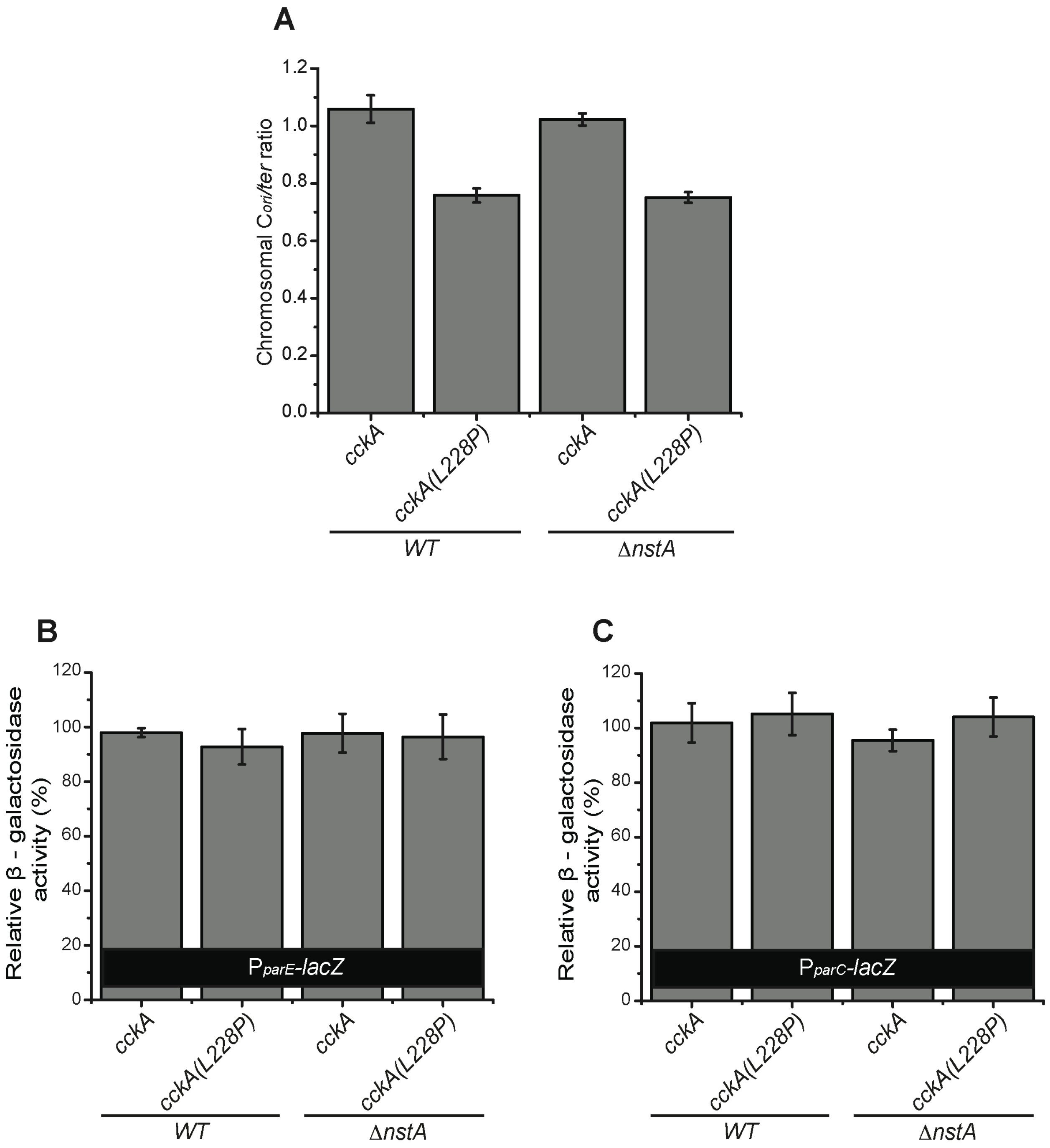

Supplementary Figure S6. (A) Relative C_{ori}/*ter* ratio obtained from qPCR on chromosomal DNA extracted from *WT, WT cckA*(L228P), Δ*nstA*, and Δ*nstA cckA*(L228P) cells. Values were normalized to a control strain, which corresponds to the *WT* strain grown in PYE medium and treated with rifampicin for three hours. Relative β-galactosidase activities of (B) P_{parE}-*lacZ* reporter, and (C) P_{parC}-*lacZ* reporter in *WT, WT cckA*(L228P), Δ*nstA*, and Δ*nstA cckA*(L228P) cells. The data represented in (A), (B) and (C) are the average of three independent experiments, ±SE.

Strain number	Mutation in CckA	Other mutations
LK128	F392L	None
LK135	D364G	CCNA_01909-(G147A)
LK137	F392L	CCNA_00364-(V208A), CCNA_01161-(G437G)
SN208	L228P	Intergenic SNP between CCNA_01536 and CCNA_01535, Intergenic SNP between CCNA_00614 and CCNA_00615.
LK124	F493C	CCNA_02093-(I457S), CCNA_02487-(G26C), CspC(A63A)
LK98	R356C	None
LK100	F496C	None
LK109	L228P	None
LK122	A317V	CspC(E54D), CCNA_03812-(A110P)

Supplementary Table S1. List of the polymorphisms identified from the nine extragenic suppressor strains that alleviated the NstADD overexpression toxicity.

Supplementary Methods

Flow cytometry analyses

Flow cytometry analyses were performed as described earlier (26). Cells were incubated at 29°C until mid-log phase, and 1 mL of culture was transferred into 9 mL of ice-cold 70% ethanol and stored overnight at −20°C for fixation. Two milliliters of the fixed cells were washed with 1 mL of staining buffer (10 mM Tris-HCl at pH 7.20, 1 mM EDTA, 50 mM sodium citrate, 0.01% Triton-X-100). The cells were then harvested by centrifugation at 8000 rpm for 5 min, and the pellet was resuspended in 1 mL of staining buffer containing 0.1 mg/mL RNase A (Roche) and incubated for 30 min at room temperature. The cells were pelleted at 8000 rpm for 5 min, and the pellet was resuspended in 1 mL of staining buffer containing 0.5 μM SYTOX green nucleic acid stain (Molecular Probes). The cells were incubated in the dark for 5 min and analyzed using an Accuri C6 flow cytometer (BD Biosciences) equipped with an argon ion laser. Relative chromosome number was directly estimated from the green fluorescence (FL1-A) value of the stained cells and analyzed using BD Accuri C6 software.

C_{ori}/*ter* ratio determination

The C_{ori}/*ter* ratios were determined as previously described (32). Cells at the mid-log phase were harvested and chromosomal DNA was extracted using DNAzol reagent and 2 μL of Ready-lyse (250U/μL). The following primer pairs were used for the qPCR reaction: Cori_fwd (5′-CGCGGAACGACCCACAAACT-3′) and Cori_rev (5′-CAGCCGACCGACCAGAGCA-3′) targeting a region close to the origin (*Cori*): Ter_fwd

(5′-CCGTACGCGACAGGGTGAAATAG-3′) and Ter_rev (5’GACGCGGCGGGCAACAT-3’) targeting a region close to the terminus (*ter*). Reactions were run using SYBR Green Supermix (Biorad) in a volume of 20 µL, containing 10 µL of supermix, 2 µL of each pair of primers (concentration 4 µM) and 8 µL of DNA on a CFX96 Real Time PCR System (Bio-Rad, CA, USA). For quantification of the results, a calibrator-normalized relative analysis was performed using CFX Manager Software for determining the relative abundance of the chromosomal C_{ori} and *ter* sites in each of the samples. The results were normalized to the C_{ori}/*ter* ratio of the wild-type control strain (NA1000) treated with rifampicin for three hours, whose C_{ori}/*ter* ratio is almost close to unity.

Strain construction

The strain **SKR1800** (*WT* + pBVMCS-4-/pMT335-P_{van}-*nstA*DD) has been previously described [1].

The various *cckA* point mutant strains namely **SN208** [Δ*nstA cckA* (L228P)], **LK98** [*WT cckA*(R356C)], **LK100** [Δ*nstA cckA*(F496C)], **LK109** [*WT cckA*(L228P)], **LK122** [*WT cckA*(A317V)], **LK124** [*WT cckA*(F493C)], **LK128** [*WT cckA*(F392L)], **LK135** [*WT cckA*(D364G)] and **LK137** [*WT cckA*(F392L)] were generated by Ultraviolet (UV) radiation based mutagenesis using *WT* or SKR1797 (Δ*nstA*) [1].

The strains namely **SN227** [Δ*nstA cckA* (L228P) + pBVMCS-4-P_{van}-*nstA*DD], **SN1140** [*WT cckA*(L228P) + pBVMCS-4-P_{van}-*nstA*DD], **SN1141** [*WT cckA*(A317V) + pBVMCS-4-P_{van}-*nstA*DD], **SN1142** [*WT cckA*(F493C) + pBVMCS-4-P_{van}-*nstA*DD], **SN1144** [*WT cckA*(F392L) + pBVMCS-4-P_{van}-*nstA*DD], **SN1145** [*WT cckA*(D364G) +

pBVMCS-4-P$_{van}$-*nstA*DD], **SN1151** [*WT cckA*(R356C) + pBVMCS-4-P$_{van}$-*nstA*DD] and **SN1152** [Δ*nstA cckA*(F496C) + pBVMCS-4-P$_{van}$-*nstA*DD] were made by electroporating pSKR126 (pBVMCS-4-*nstA*DD) [1] into SN208 [Δ*nstA cckA* (L228P)], LK109 [*WT cckA*(L228P)], LK122 [*WT cckA*(A317V)], LK124 [*WT cckA*(F493C)], LK128 [*WT cckA*(F392L)], LK135 [*WT cckA*(D364G)], LK98 [*WT cckA*(R356C)] and LK100 [Δ*nstA cckA*(F496C)] respectively,

The strains **SN377** (*WT; xylX*::P$_{xyl}$-*gfp-parB*), **SN379** [Δ*nstA cckA*(L228P); *xylX::P$_{xyl}$-gfp-parB*] and **SN559** (Δ*nstA; xylX*::P$_{xyl}$-*gfp-parB*) were made by electroporating pSN190 (pXGFP4C1-P$_{xyl}$-*gfp-parB*) into *WT,* SN208 and SKR1797 respectively.

The strains **SN461** [Δ*nstA cckA*(L228P) + pJSX-*dnaA*], **SN465** [Δ*nstA cckA*(L228P) + pJSX-*dnaA* (R357A)] and **SN467** [Δ*nstA cckA*(L228P)+pJS14] were made by electroporating pJSX-*dnaA*, pJSX-*dnaA* (R357A) [2] and pJS14 [3] into SN208 respectively.

The strains **SN505** (*WT*; *xylX::* P$_{xyl}$-*gfp-parB* + pBVMCS-4) and **SN1153** (*WT*; *xylX::*P$_{xyl}$-*gfp-parB* + pBVMCS-4-P$_{van}$-*nstA*DD) were made by electroporating pBVMCS-4 [4] and pSKR126 into the strain SN377 respectively.

The strains **SN740** (*WT* + pLac290-P$_{pilA}$-*lacZ*), **SN742** (Δ*nstA* + pLac290-P$_{pilA}$-*lacZ*) and **SN744** [Δ*nstA cckA*(L228P) + pLac290-P$_{pilA}$-*lacZ*] were made by electroporating pJS70 (pLac290-P$_{pilA}$-*lacZ*) [5] into *WT,* SKR1797 and SN208 respectively.

The strains **SN741** (*WT* + pLac290-P$_{tacA}$-*lacZ*), **SN743** (Δ*nstA* + pLac290-P$_{tacA}$-*lacZ*) and **SN745** [Δ*nstA cckA*(L228P) + pLac290-P$_{tacA}$-*lacZ*] were made by electroporating pMV05 (pLac290-P$_{tacA}$-*lacZ*) [6] into *WT,* SKR1797 and SN208 respectively.

The strains **SN1361** (*WT* + pLac290-P$_{fliM}$-*lacZ*), **SN1365** (Δ*nstA* + pLac290-P$_{fliM}$-*lacZ*) and **SN1369** [Δ*nstA cckA*(L228P) + pLac290-P$_{fliM}$-*lacZ*] were made by electroporating pLac290-P$_{fliM}$-*lacZ* into *WT,* SKR1797 and SN208 respectively.

The strains **SN1406** (*WT* + pLac290-P$_{flbT}$-*lacZ*), **SN1407** (Δ*nstA* + pLac290-P$_{flbT}$-*lacZ*) and **SN1408** [Δ*nstA cckA*(L228P) + pLac290-P$_{flbT}$-*lacZ*] were made by electroporating pLac290-P$_{flbT}$-*lacZ* into *WT,* SKR1797 and SN208 respectively.

The *cckA*(L228P) back cross strains **SN739** [*WT*;*ΔcckA*(L228P)] **SN769** [*ΔnstA*; *cckA*(L228P)] was made by backcrossing the *cckA*(L228P) point mutation in SN208 into *WT* and SKR1797. pSN155 (pNPTS-*cckA* backcross construct) was used to transform SN208 and the transformants were plated on PYE supplemented with Kanamycin. Further øCr30 lysates of the transformants were made and was used for transducing into *WT* and SKR1797 thereby generating SN 739 and SN769. The backcross strain, SN769, was electroporated with pSKR126, to obtain **SN771** [*ΔnstA*; *cckA*(L228P) + pBVMCS-4-P$_{van}$-*nstA*DD].

The strains **SN1435** [*WT cckA*(L228P) + pLac290-P$_{pilA}$-*lacZ*], **SN1436** [*WT cckA*(L228P) + pLac290-P$_{tacA}$-*lacZ*], **SN1437** [*WT cckA*(L228P) + pLac290-P$_{fliM}$-*lacZ*] and **SN1438** [*WT cckA*(L228P) + pLac290-P$_{flbT}$-*lacZ*) were made by electroporating pJS70, pMV05, pLac290-P$_{fliM}$-*lacZ* and pLac290-P$_{flbT}$-*lacZ* into SN739.

The strains **SN1439** (*WT* + pLac290-P$_{parC}$-*lacZ*), **SN1441** (Δ*nstA* + pLac290-P$_{parC}$-*lacZ*), **SN1443** [Δ*nstA cckA*(L228P) + pLac290-P$_{parC}$-*lacZ*] and **SN1445** [*WT cckA*(L228P) + pLac290-P$_{parC}$-*lacZ*], were made by electroporating pLac290-P$_{parC}$-*lacZ* into *WT,* SKR1797, SN208 and SN739 respectively.

The strains **SN1447** (*WT* + pLac290-P$_{parE}$-*lacZ*), **SN1449** (Δ*nstA* + pLac290-P$_{parE}$-*lacZ*), **SN1451** [Δ*nstA cckA*(L228P) + pLac290-P$_{parE}$-*lacZ*] and **SN1453** [*WT cckA*(L228P) + pLac290-P$_{parE}$-*lacZ*], were made by electroporating pLac290-P$_{parE}$-*lacZ* into *WT,* SKR1797, SN208 and SN739 respectively.

Plasmid construction

The Plasmid **pBVMCS-4-P$_{van}$-*nstA*DD** is the same as **pSKR126** (pMT335-P$_{van}$-*nstA*DD) described previously [1]. This was made by PCR amplifying *nstADD* (in which the C-terminal Ala-Ala codons were replaced with Asp-Asp [GAU, GAC] codons) and cleaving the fragment with *Nde*I and *Eco*RI and ligating into *Nde*I/*Eco*RI treated pBVMCS-4 [4].

The plasmid **pSN155** (pNPTS-*cckA*-backcross) construct was made by PCR amplifying a region 750bp upstream of *cckA.* The PCR fragment was digested with *Eco*RI/*Hind*III. The digested fragment was ligated into pNPTS138 (M.R.K Alley, unpublished) cut with *Eco*RI/*Hind*III.

Plasmid **pSN190** (pXGFP4-C1-P$_{xyl}$-*gfp-parB*) was made by PCR amplifying *parB* and cleaving it with *Bgl*II/*Eco*RI, wherein the predicted start codon ATG was replaced

with GTG that carried an overlapping *Bgl*II recognition site to allow proper placement of *parB* facilitating N-terminal GFP fusion. The alleles were ligated into pXGFP4-C1 vector (M.R.K Alley unpublished) cut with *Bgl*II/*Eco*RI.

To make **pSN206** (P_{fliM}-*lacZ*, a kind gift from Patrick Viollier) nucleotides 2298862-2299971 of NA1000 genome (CP001340) was amplified and ligated as *Eco*RI/*Hind*III fragment into a medium-copy plasmid pJGZ290 [7] to drive the transcription of the promoterless *lacZ* gene.

Plasmid **pSN209** (P_{flbT}-*lacZ,* a kind gift from Patrick Viollier) was made by amplifying nucleotides 1633750-1634327 of the NA1000 genome (CP001340) and ligated as *Eco*RI/*Hind*III fragment into the medium-copy plasmid pJGZ290 [7] to drive the transcription of the promoterless *lacZ* gene.

Plasmid **pSN210** (pLac290-P_{parC}-*lacZ*) was made by amplifying nucleotides 1755523-1755922 of the NA1000 genome (CP001340) and ligated as *Eco*RI/*Hind*III fragment into the medium-copy plasmid pJGZ290 [7] to drive the transcription of the promoterless *lacZ* gene.

Plasmid **pSN211** (pLac290-P_{parE}-*lacZ*) was made by amplifying nucleotides 2199977-2200492 of the NA1000 genome (CP001340) and ligated as *Eco*RI/*Hind*III fragment into the medium-copy plasmid pJGZ290 [7] to drive the transcription of the promoterless *lacZ* gene.

Plasmids pJSX-*dnaA*, pJSX-*dnaA* (R357A) and pJS14 were obtained from Justine Collier and are described previously [2, 3].

References

[1] S. Narayanan, B. Janakiraman, L. Kumar, S.K. Radhakrishnan, A cell cycle-controlled redox switch regulates the topoisomerase IV activity, Genes Dev, 29 (2015) 1175-1187.
[2] C. Fernandez-Fernandez, D. Gonzalez, J. Collier, Regulation of the activity of the dual-function DnaA protein in Caulobacter crescentus, PLoS One, 6 (2011) e26028.
[3] K.C. Quon, G.T. Marczynski, L. Shapiro, Cell cycle control by an essential bacterial two-component signal transduction protein, Cell, 84 (1996) 83-93.
[4] M. Thanbichler, A.A. Iniesta, L. Shapiro, A comprehensive set of plasmids for vanillate- and xylose-inducible gene expression in Caulobacter crescentus, Nucleic Acids Res, 35 (2007) e137.
[5] J.M. Skerker, L. Shapiro, Identification and cell cycle control of a novel pilus system in *Caulobacter crescentus*, Embo J, 19 (2000) 3223-3234.
[6] M.V. Marques, S.L. Gomes, J.W. Gober, A gene coding for a putative sigma 54 activator is developmentally regulated in Caulobacter crescentus, J Bacteriol, 179 (1997) 5502-5510.
[7] G. Ditta, S. Stanfield, D. Corbin, D.R. Helinski, Broad host range DNA cloning system for gram-negative bacteria: construction of a gene bank of Rhizobium meliloti, Proc Natl Acad Sci U S A, 77 (1980) 7347-7351.
